# Dietary polyphenols and sarcopenia: epigenetic mechanisms and geroscience perspectives for muscle health in aging

**DOI:** 10.3389/fragi.2025.1696473

**Published:** 2026-01-06

**Authors:** Guilherme Da Silva Rodrigues, Leonardo Santos Lopes da Silva, Andressa Crystine da Silva Sobrinho, Jonas Benjamim, Gabriela Ferreira Abud, Gabriela Ueta Ortiz, Ellen Cristini de Freitas, Carlos Roberto Bueno Júnior

**Affiliations:** 1 Department of Internal Medicine, School of Medicine of Ribeirão Preto, University of São Paulo, Ribeirão Preto, Brazil; 2 Institute for Physical Activity and Nutrition (IPAN), School of Exercise and Nutrition Sciences, Deakin University, Geelong, VIC, Australia; 3 Department of Health Sciences, School of Medicine of Ribeirão Preto, University of São Paulo, Ribeirão Preto, Brazil; 4 School of Physical Education and Sport of Ribeirão Preto, University of São Paulo (USP), Ribeirão Preto, Brazil

**Keywords:** epigenetics, muscle aging, nutrigenomics, polyphenols, sarcopenia

## Abstract

This review explores the effects of dietary polyphenols, such as resveratrol, quercetin, epigallocatechin gallate, and curcumin, on sarcopenia, with a particular focus on the underlying molecular and epigenetic mechanisms. These bioactive compounds may modulate key signaling pathways, including mTOR, NF-κB, and AMPK, while also influencing epigenetic processes such as DNA methylation, histone modifications, and microRNA regulation. Through these actions, polyphenols may reduce oxidative stress and chronic low-grade inflammation (inflammaging), enhance mitochondrial function, and contribute to the preservation of muscle mass and strength in older adults. Evidence from experimental and clinical studies investigating the impact of polyphenols on muscle health and their potential in the prevention or attenuation of sarcopenia will be discussed. In addition, current challenges and future perspectives will be addressed, emphasizing the role of epigenetic biomarkers and the potential synergy with physical exercise as part of integrated geroscience strategies to optimize muscle health during aging.

## 1 Introdution

Aging is accompanied by an interaction between environmental and physiological factors that favors the development of sarcopenia. In addition to lifestyle-related factors such as reduced physical activity and inadequate nutrient intake, skeletal muscle undergoes measurable structural and metabolic decline beginning in the third decade of life (Evans and Lexell, 1995), and continues to be progressively affected during aging by several biological hallmarks of aging, including epigenetic alterations such as DNA methylation drift, histone modifications, and dysregulated non-coding RNAs, which compromise its ability to regenerate and maintain functionality (Daily and Park, 2022; Wang et al., 2022). Among these, mitochondrial dysfunction, oxidative stress, epigenetic instability, and low-grade chronic inflammation stand out. Mitochondrial dysfunction impairs energy production and promotes the accumulation of reactive oxygen species (ROS), which exceed the endogenous antioxidant capacity and cause cellular damage and activation of inflammatory pathways (Napolitano et al., 2021). Systemic inflammation impairs the signaling of key anabolic pathways, such as IGF-1 and insulin, contributing to reduced protein synthesis (López-Otín et al., 2023).

Furthermore, aging is associated with anabolic resistance, characterized by a blunted response to amino acid intake and physical exercise, even under normally stimulatory conditions. Neuromuscular dysfunction, marked by the loss of alpha motor neurons and the deterioration of motor unit integrity, reduces the neural stimulus required to maintain muscle mass (Borzuola et al., 2020). The infiltration of fat into muscle tissue and increased fibrosis contribute to the decline in muscle quality, while a reduction in the number and function of satellite cells limits muscle regeneration. These multiple factors act synergistically, promoting a persistent negative protein balance that ultimately leads to the progressive loss of muscle mass and function observed in age-related sarcopenia (Chinvattanachot et al., 2024).

Given the role of inflammation and oxidative stress in the pathophysiology of sarcopenia, interventions targeting these deleterious effects of aging have emerged as promising therapeutic and prevention strategies within a multidisciplinary care framework (Hu et al., 2024). Dietary polyphenols are bioactive compounds present in plant-based foods. They are named for containing two or more phenolic hydroxyl groups. Polyphenols are broadly classified into two main categories based on their chemical structure: flavonoids and non-flavonoids. Flavonoids are further subdivided into six subclasses: (1) flavanones; (2) flavonols; (3) flavones; (4) isoflavones; (5) anthocyanins; and (6) flavan-3-ols—according to the number and type of linkages between their phenolic groups. Non-flavonoid polyphenols primarily include phenolic acids, stilbenes, lignans, coumarins, and tannins (Medoro et al., 2024).

The primary dietary sources of polyphenols are fruits and vegetables; however, their bioavailability is influenced by numerous factors. These include environmental conditions, food processing methods, gastrointestinal metabolism, particularly the action of the gut microbiota, and cellular metabolic pathways. Such factors can significantly affect the absorption, distribution, and biological activity of polyphenols within the human body (Arfaoui, 2021).

The current review aims to elucidate the potential of dietary polyphenols as a therapeutic strategy for sarcopenia by exploring the underlying molecular and cellular mechanisms. Additionally, it discusses their relevance to muscle health during aging and the challenges associated with their implementation in clinical practice.

## 2 Definition, diagnostic criteria, and clinical relevance of sarcopenia

According to the revised criteria of the EWGSOP2 (2019), the contemporary definition of this condition transcends the mere quantitative decrease in muscle tissue and necessarily includes a qualitative assessment that encompasses significant muscle strength loss and a marked deterioration in physical performance, particularly in the advanced stages of the disease (Sayer and Cruz-Jentoft, 2022). EWGSOP2 further categorizes sarcopenia into probable, confirmed, and severe stages, based on progressive impairments in muscle strength, mass, and physical performance.

Early identification of sarcopenia is critical for effective management, requiring the use of standardized diagnostic criteria and reliable objective assessments (Liu et al., 2024; Voulgaridou et al., 2024). Among the techniques employed, dual-energy X-ray absorptiometry (DXA) stands out for its precision in measuring skeletal muscle mass, while handgrip dynamometry represents a practical and efficient method for assessing muscle strength through handgrip strength (Liu et al., 2024; Ooi and Welch, 2024). Furthermore, functional tests such as gait speed and the chair stand test are commonly applied to evaluate overall physical performance and to detect early declines in functionality typically associated with sarcopenia (Liu et al., 2024; Voulgaridou et al., 2024).

Recent advances have explored the use of circulating biomarkers as potential complementary tools for the early diagnosis of sarcopenia; however, there is still no consensus regarding the most effective biomarkers (Veronesi et al., 2024; Lian et al., 2024). Examples include myostatin, C-reactive protein (CRP), and interleukin-6 (IL-6), although consensus is lacking on their clinical applicability. Concurrently, significant challenges persist, including the lack of universal diagnostic criteria and variability in the measurement techniques and instruments employed. To overcome these challenges, it is essential to adopt a multidimensional approach that integrates the assessment of muscle mass, strength, and physical performance, utilizing standardized methods and continuously investing in research on new biomarkers (Pedauyé-Rueda et al., 2024). This integrated strategy can substantially enhance diagnostic accuracy and promote more effective and timely interventions, ultimately improving the quality of life for older population.

### 2.1 Clinical, functional, and psychosocial impact of sarcopenia

The clinical impact of sarcopenia on older adults is broad and multifaceted, encompassing both direct health complications and indirect consequences that affect the prognosis of various clinical conditions (He et al., 2021; Xue et al., 2021). Aging is strongly associated with an increased prevalence of cardiovascular diseases, type 2 diabetes, and postoperative complications, leading to more frequent and prolonged hospitalizations, as well as severe complications such as hospital-acquired infections and higher mortality rates across diverse clinical settings, including oncology patients (Li et al., 2023; Wang H. et al., 2021). Although these conditions are strongly influenced by aging, the presence of sarcopenia further exacerbates clinical vulnerability, acting as an aggravating factor for multiple diseases and significantly compromising recovery and survival among older patients (Sun et al., 2022).

In addition to its impact on skeletal muscle, physiological aging induces progressive degenerative changes across the entire musculoskeletal system (Boros and Freemont, 2017). These alterations include reductions in bone mineral density that contribute to osteoporosis, as well as structural and inflammatory changes within joint tissues that promote the development of osteoarthritis (Goldring and Goldring, 2016). Together, these age-related impairments in bone and joint integrity interact with declining muscle mass and strength, compounding the risk of mobility limitations, chronic pain, falls, and functional decline (Wang et al., 2025). By understanding these interconnected processes, the multifactorial nature of musculoskeletal deterioration during senescence becomes clearer, contextualizing sarcopenia as part of a broader trajectory of tissue degeneration rather than an isolated condition (Boccardi, 2024; Larsson et al., 2019).

From a functional perspective, sarcopenia triggers a marked decline in physical capacity, particularly in mobility and postural stability, substantially increasing the risk of falls and bone fractures (Yuan and Larsson, 2023; Su et al., 2022). These are leading contributors to morbidity and mortality among older adults. This reduction in functional capacity severely compromises the autonomy of older adults, limiting their ability to independently perform basic and instrumental activities of daily living, such as dressing, eating, managing medications, and carrying out simple household tasks (Liu et al., 2023). This growing functional dependence considerably diminishes quality of life and intensifies the use of healthcare and social support services, representing a significant burden for both individuals and their families and caregivers (Izzo et al., 2021).

Moreover, the psychosocial repercussions of sarcopenia are substantial, often manifesting through social isolation, depression, and anxiety, which may be exacerbated by the perception of frailty and the progressive loss of independence (Yuan and Larsson, 2023). These psychological implications not only compromise the emotional wellbeing of older adults but also have the potential to further worsen clinical and functional outcomes, creating a vicious cycle in which physical and psychological deteriorations mutually reinforce one another (Yuan and Larsson, 2023; Chen and Wu, 2025). This reciprocal deterioration is often intertwined with frailty syndrome, further increasing vulnerability to adverse health outcomes. Therefore, a comprehensive approach to sarcopenia must incorporate integrated strategies encompassing clinical, functional, and psychosocial interventions to promote a better and more sustainable quality of life for the older population.

## 3 Biological, molecular and epigenetics factors involved in sarcopenia

Sarcopenia is a multifactorial condition characterized by the progressive decline in muscle mass, strength, and function with aging. Several biological and molecular factors contribute to its onset, including impaired muscle regeneration, chronic low-grade inflammation, oxidative stress, and metabolic dysfunction (He et al., 2020).

From a genetic perspective, polymorphisms in genes associated with muscle mass and function have been linked to sarcopenia risk. However, genetic factors alone do not fully explain the large interindividual variability, highlighting the crucial role of epigenetic mechanisms (Antoun et al., 2022).

Recent epigenome-wide studies demonstrate that DNA methylation is a key regulator of genes involved in muscle metabolism, myoblast differentiation, and regenerative capacity. Specific methylation signatures have been associated with appendicular lean mass, grip strength, and gait speed (Antoun et al., 2022). Differentially methylated regions are enriched in genes related to myotube fusion, oxidative phosphorylation, and voltage-gated calcium channels, indicating that epigenetic modifications affect essential pathways in muscle homeostasis.

Moreover, DNA methylation of secretory muscle factors, such as myokines, has been identified as a determinant of muscle function and a potential biomarker for early detection of sarcopenia (Li et al., 2024). This emphasizes the value of epigenetic profiling of muscle-derived secretions for diagnostic and therapeutic strategies.

Immunological mechanisms also intersect with epigenetic regulation in sarcopenia. Persistent activation of innate and adaptive immune pathways may alter DNA methylation landscapes of immune cells infiltrating skeletal muscle, thereby contributing to chronic inflammation and accelerating muscle decline (Bogucka et al., 2025; Yedigaryan et al., 2022).

Finally, combining genetic and epigenetic scores has shown greater predictive power for sarcopenia risk compared with genetic variants alone, reinforcing the role of epigenetics as a mediator between genetic predisposition and environmental exposures (He et al., 2020).

**FIGURE 1:**
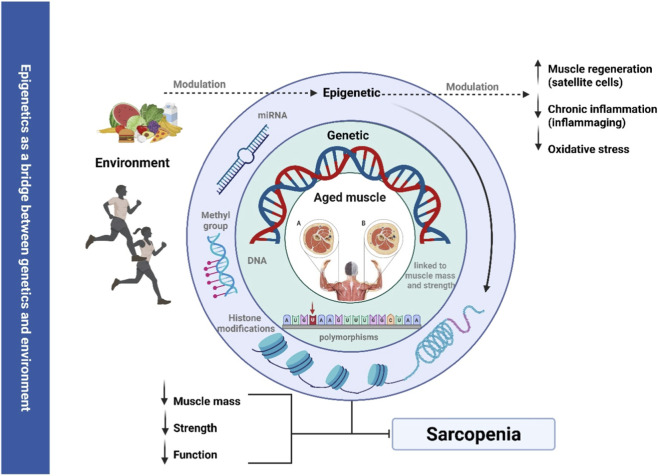
Epigenetics is a bridge between genetics and environment in the context of sarcopenia. Note Environmental factors, such as physical exercise and dietary intake, modulate epigenetic mechanisms (DNA methylation, histone modifications, and microRNAs), which in turn influence genetic regulation of muscle mass and strength. These epigenetic changes affect key biological processes, including satellite cell mediated muscle regeneration, chronic low-grade inflammation (inflammaging), and oxidative stress. The central panel illustrates muscle morphology: A: represents young muscle, while; B: represents aged muscle characterized by atrophy and impaired function. Collectively, these interactions contribute to reduced muscle mass, strength, and function, ultimately leading to sarcopenia.

Together, these findings suggest that classical biological and molecular factors of sarcopenia should be reinterpreted through an epigenetic lens, positioning epigenetic regulation as a central link between genetics, environment, and muscle aging (Figure 1).

## 4 Dietary polyphenols: properties and sources

Polyphenols are naturally occurring phytonutrients in plant-based foods and have significant antioxidant and anti-inflammatory effects on the human body (Kesh et al., 2023). These compounds provide health benefits through their high antioxidant capacity, which allows them to neutralize reactive oxygen species and modulate gene expression (Fagbohun et al., 2023). The benefits of a diet rich in polyphenols are linked to the prevention of cardiovascular and neurodegenerative diseases, cancer, and an important role in longevity (Rudrapal et al., 2022), in addition to reducing oxidative stress, which is one of the main factors in aging (Hajam et al., 2022).

There are a variety of polyphenols, totaling approximately 8,000 types (Beylerli et al., 2022). This range is divided into groups such as flavonoids, lignans, tannins, phenolic acids, and stilbenes (Ciupei et al., 2024). Despite their diversity, they share antioxidant and anti-inflammatory properties that contribute to cardiovascular and brain health (Olufunmilayo et al., 2023; Rana et al., 2022).

Regular intake of foods rich in polyphenols is associated with several benefits, such as reduced blood pressure and improved lipid profile (Rana et al., 2022; Hu, 2024). A study carried out by Karam et al., (2018) with 211 participants, aged between 50 and 80 years, used two non-consecutive 24-h recalls to estimate polyphenol intake, and the findings showed that residents of Mallorca in Spain had red wine as one of the main sources of polyphenols, and that factors such as gender, educational level, income, alcohol consumption and physical activity influenced the intake of these bioactive compounds (Karam et al., 2018). The average daily intake of polyphenols among adults and older adults in Mallorca, Spain, was 332.7 mg/day, with a median of 299 mg/day (Karam et al., 2018).

A study conducted by Miranda et al. (2016), in the city of São Paulo, Brazil, with 1,103 participants, including both adults and older individuals, used the food frequency questionnaire (FFQ) to assess the intake of foods rich in polyphenols. The results showed that the average daily intake of polyphenols was 337.5 mg/day, with coffee being the main source in this sample, contributing with 70.5% of the total intake, but highlighting other significant sources such as citrus fruits (4.6%) and tropical fruits (3.4%) (Miranda et al., 2016). Another interesting finding of the study is that older people had a significantly higher intake of polyphenols compared to younger adults (Miranda et al., 2016) (Table 1). These findings underscore how dietary patterns and cultural factors influence the primary sources and intake levels of polyphenols in different populations.

**TABLE 1:** Summary of polyphenol intake and dietary sources in various populations.

PMID	Polyphenol intake (mg/day)	Main sources	MCA	Target population (age)	Main findings
21490142 (Pérez-Jiménez et al., 2011)	1,193 ± 510 mg/day (average), 820 ± 335 mg/day (aglycone equivalents)	Fruits, vegetables, red wine, dark chocolate	24h-DR	4,942 French adults (45–60 years)	The average polyphenol intake was 1,193 ± 510 mg/day, with significant variation between individuals. Main sources: fruits, vegetables, red wine, and dark chocolate
24606711 (González et al., 2013)	332.7 mg/day (average)	Red wine, fruits (citrus, tropical)	FFQ	159 older adults (68 men, 91 women)	Flavonoid intake was inversely associated with plasma malondialdeído, indicating an impact on lipid peroxidation
26810764 (Miranda et al., 2016)	377.5 mg/day (average)	Coffee, citrus fruits, tropical fruits	FFQ	1,103 adults and older adults (age unspecified)	Coffee was the main source of polyphenols, with significant differences between adults and older adults
29381732 (Karam et al., 2018)	332.7 mg/day	Alcoholic beverages (mainly red wine), fruits, vegetables	24h-DR	211 older adults from Mallorca (50–80 years)	Polyphenol intake was dominated by alcoholic beverages, especially red wine

MCA, method of consumption assessment; FFQ, Semi-quantitative food frequency questionnaire; 24h FFQ, 24-h food questionnaire.

The following table concisely summarizes the main properties and health benefits of the most common polyphenols, such as catechins, resveratrol, curcumin, quercetin and epigallocatechin gallate (Table 2). We have included examples of foods that present a risk to these compounds, in addition to their effects on human health.

**TABLE 2:** Properties and benefits of polyphenols.

PMID	Property	Polyphenol examples	Polyphenol effects	Health benefits
32143309 (Musial et al., 2020)	Antioxidant Properties	Catechins (green tea), Resveratrol (red wine)	Neutralizes free radicals, reduces oxidative stress, anti-inflammatory	Prevents cardiovascular diseases, cancer, and neurodegenerative diseases by reducing oxidative damage
30816367 (Galiniak et al., 2019)	Anti-inflammatory Properties	Resveratrol (red wine), Curcumin (turmeric)	Reduces inflammation, supports heart health, antioxidant	Reduces the risk of cardiovascular diseases, diabetes type 2, neurodegenerative diseases, and arthritis
17569207 (Menon and Sudheer, 2007)	Oxidative Stress Modulating Properties	Quercetin (apples, onions, grapes)	Anti-inflammatory, antioxidant, neuroprotective	Protects cells from oxidative stress and improves antioxidant defense system function
37513932 (Aghababaei and Hadidi, 2023)	Cardiovascular Health	Resveratrol (red wine, grapes), Curcumin (turmeric)	Reduces oxidative stress, anti-inflammatory, immune support	Reduces the risk of cardiovascular diseases and improves vascular health
39942757 (Capasso et al., 2025)	Brain Health	Resveratrol (red wine, grapes), Curcumin (turmeric)	Antioxidant, anti-inflammatory, metabolic support	Protects the brain from oxidative stress and inflammation, contributing to neuroprotection
37513932 (Aghababaei and Hadidi, 2023)	Inflammation Control	Resveratrol (red wine, grapes), Curcumin (turmeric)	Reduces inflammatory mediator production and decreases pro-inflammatory cytokines	Relieves chronic inflammatory conditions, such as arthritis and type 2 diabetes
30816367 (Galiniak et al., 2019)	Cancer Prevention	Resveratrol (red wine, grapes), Quercetin (apples, onions)	Inhibits the growth of tumor cells and induces apoptosis (programmed cell death)	May help prevent cancer by inhibiting growth and promoting death of cancerous cells

The interesting thing about natural foods is that, in some cases, they appear in more than one category due to their variety of bioactive compounds, so it is important to have a rich and diverse diet (Table 2).

### 4.1 Polyphenols in bone and joint health

Bone is a metabolically active tissue that undergoes continuous remodeling through the coordinated actions of osteoblasts, which form new bones, and osteoclasts, which resorb mineralized matrix. With aging, this coupling becomes progressively unbalanced, with a relative increase in resorption and a decline in bone formation, contributing to bone loss, microarchitectural deterioration, and increased fracture risk, particularly in postmenopausal osteoporosis. In this context, dietary polyphenols have emerged as promising adjuncts for skeletal health, since experimental and clinical studies suggest that they can modulate oxidative stress, inflammatory signaling, and key molecular pathways that regulate osteoblast and osteoclast function, thereby influencing bone remodeling and osteoporosis progression (Perrone et al., 2025).

At the cellular level, several polyphenols appear to promote an osteoanabolic profile by stimulating osteoblast proliferation, differentiation, and mineralization, while simultaneously attenuating osteoclastogenesis and bone resorption. Mechanistic studies indicate that these effects involve the regulation of osteogenic pathways such as Wnt/β-catenin and MAPK, as well as modulation of the RANKL/OPG axis and other signaling nodes that couple bone formation and resorption (Perrone et al., 2025; Li et al., 2022). In preclinical models, resveratrol is one of the best characterized compounds, showing the capacity to improve bone microarchitecture and turnover markers in aging-related osteoporosis. These benefits have been linked to an upregulation of FoxO1 and SIRT1 expression and a more favorable RANKL/OPG balance in bone tissue, supporting the idea that polyphenols can act on both oxidative and inflammatory pathways and directly on gene networks that control osteoblast and osteoclast activity (Ameen et al., 2020).

Beyond bone mass, polyphenols may also contribute to joint health. Osteoarthritis is characterized by progressive degeneration of articular cartilage, subchondral bone remodeling, and synovial inflammation, processes in which oxidative stress and pro-inflammatory cytokines play central roles. Experimental and translational studies have shown that different polyphenolic compounds, including flavonoids and stilbenes, can reduce the production of inflammatory mediators, downregulate matrix-degrading enzymes, and mitigate oxidative damage in chondrocytes and synovial cells, ultimately preserving cartilage matrix and improving functional outcomes in osteoarthritis models (Ansari et al., 2020; Sirše, 2022). Together, these findings suggest that polyphenols may exert osteoprotective and chondroprotective effects by acting on shared molecular pathways that link inflammation, redox imbalance, and tissue remodeling in bone and joint structures.

## 5 Molecular and epigenetic mechanisms of polyphenols in sarcopenia

### 5.1 Quercetin and resveratrol in mTOR/AMPK/NF-κB pathways

The Mechanistic Target of Rapamycin (mTOR), Nuclear Factor Kappa-light-chain-enhancer of activated B cells (NF-κB), and AMP-activated protein kinase (AMPK) signaling pathways are interconnected; dysregulation of these pathways contributes to age-related muscle atrophy, whereas their appropriate modulation by exercise supports muscle mass maintenance and may help prevent sarcopenia (Li et al., 1998; Cai et al., 2004; Zeng et al., 2020; Tang et al., 2019). mTOR hyperactivity can inhibit autophagy, while NF-kB activation induces inflammation and protein degradation (Cai et al., 2004; Castets et al., 2013; Li and Reid, 2000). mTOR and NF-kB act synergistically in muscle degeneration, and this action creates a vicious cycle that accelerates muscle loss during aging (Cai et al., 2004; Tang et al., 2019; Lee and Goldberg, 2015).

AMPK activity counteracts the negative effects of mTOR and NF-kB (Wang et al., 2020). When activated by polyphenols, it inhibits NF-kB–induced inflammation and promotes mitochondrial regeneration, supporting autophagy and delaying sarcopenia progression (Don et al., 2022). Another key point about polyphenols is that their consumption directly acts on gene expression through epigenetic mechanisms, including DNA methylation, histone acetylation/deacetylation, and regulation of non-coding RNAs (Číž et al., 2020), thereby influencing genes related to autophagy, mitochondrial biogenesis, and inflammatory responses (Abdul et al., 2017; Ayissi et al., 2014). These epigenetic effects resulting from the consumption of polyphenols not only regulate cell proliferation and the stress response but also manage to amplify mechanisms related to the regeneration and preservation of muscle mass, mitigating the effects of sarcopenia (Russo et al., 2017).

Quercetin is a flavonoid present in several easily accessible foods in diets, the most common foods being apples, onions, grapes, green tea and berries (Guven et al., 2019). Its consumption, when associated with signaling pathways and muscle health, presents several benefits for the aging process. Resveratrol is one of the most studied polyphenols for presenting beneficial effects on aging and general health (Galiniak et al., 2019).

Resveratrol and quercetin have synergistic effects and when used together, they show a positive effect in the modulation of muscle health (Rayalam et al., 2011). When combined these compounds can simultaneously activate the AMPK e Sirt1 pathways, while negatively regulating mTOR and NF–kB signaling (Giovannini and Bianchi, 2017). The combined effects are particularly useful for preserving muscle health during aging, with the maintenance of muscle mass (Pan et al., 2025). In the case of resveratrol in moderate doses (∼24 mg/kg of body weight), it already has positive actions in influencing cell signaling (Shaito et al., 2020). Protective functions on muscle health are caused by the consumption of resveratrol and quercetin (Salucci and Falcieri, 2020).

These polyphenols act on muscle health by negatively regulating the mTOR pathway, promoting autophagy, increasing mitochondrial biogenesis and reducing inflammation (Lin et al., 2024). These benefits have a positive influence on the aging process and preservation of muscle mass, making resveratrol a promising ally for maintaining muscle function, preventing sarcopenia and improving metabolic health (Ispoglou et al., 2023; Micheli et al., 2023).

When we turn our attention to resveratrol and quercetin in the activation of NF-kB, the literature shows an inhibition in the activation of NF-Kb in several experimental models (Das et al., 2022; Farkhondeh et al., 2020; Yang et al., 2022), which leads to a reduction in the production of pro-inflammatory cytokines such as TNF-α, IL-6 and IL-8 (Palacz-Wrobel et al., 2017; Bisht et al., 2010). Reducing inflammation by inhibiting NF-Kb using resveratrol helps protect muscles from chronic damage and age-related muscle atrophy, maintaining the integrity of muscle cells and promoting muscle regeneration (Pan et al., 2025; Nosaka et al., 2024).

Associated with the AMPK pathway, resveratrol and quercetin help play a crucial role in regulating cellular metabolism, improving mitochondrial function (Wang M. et al., 2021). This process is important for muscle health, since mitochondria are essential for supplying energy to muscle cells during the aging process. Another benefit of resveratrol for signaling pathways is the increased expression of PGC-1α, a key regulator of mitochondrial biogenesis, promoting greater ATP production, which positively favors muscle endurance and recovery, especially when focused on aging (Gurd, 2011; Lagouge et al., 2006).

### 5.2 Polyphenols in inflammaging and oxidative stress

When lifestyle patterns cause persistent activation of NF-kB, systemic inflammation arises (Bosma-den Boer et al., 2012; Kannan et al., 2025; Klimczak and Śliwińska, 2024) promoting cellular dysfunction and impairing muscle regeneration (Londhe and Guttridge, 2015; Chazaud, 2016). Polyphenols such as resveratrol, quercetin and curcumin have potent anti-inflammatory properties, and when consumed, they are capable of modulating levels of NF-kB activation, alleviating the expression of pro-inflammatory cytokines (such as TNF-α, IL-6 and IL-1β) (Jantan et al., 2021).

This process helps to reduce chronic inflammation, preserving muscle function and preventing the process of sarcopenia (Dalle et al., 2017; Jensen, 2008). Another positive aspect of these polyphenolic compounds is that they modulate the production of reactive oxygen species (ROS), significantly reducing the oxidative stress generated by inflammation, minimizing the cellular damage caused by inflammation (Rudrapal et al., 2022; Zhang and Tsao, 2016; Mileo et al., 2016).

Oxidative stress occurs when there is an imbalance between the production of free radicals and the capacity to neutralize them through endogenous antioxidant systems (Hassan et al., 2024). Oxidative stress contributes detrimentally to cellular damage, including muscle damage, impairing mitochondrial function and accelerating the muscle aging process (Chen et al., 2023; Xu and Wen, 2023). Mitochondrial function plays an important role in maintaining muscle health, especially in older adults (Chen et al., 2023; Sligar et al., 2022).

Mitochondria are responsible for the production of cellular energy, essential for muscle contraction and cell regeneration (Casanova et al., 2023). Polyphenols such as resveratrol and quercetin have actions in the body to increase mitochondrial biogenesis, a process in which new mitochondria are formed, improving the energy function of muscle cells (Chodari et al., 2021; Yadav et al., 2022).

Polyphenols such as epigallocatechin gallate, resveratrol and curcumin have powerful antioxidant properties that can neutralize free radicals, increasing the activity of antioxidant enzymes such as superoxide dismutase, catalase and glutathione peroxidase, which act to protect muscle cells against oxidative damage (Rudrapal et al., 2022; Jena et al., 2023).

### 5.3 Epigenetic regulation of muscle health by polyphenols

Epigenetic mechanisms including DNA methylation, histone modifications, and non-coding RNAs play a central role in the regulation of muscle plasticity, repair, and aging. These processes are dynamically influenced by environmental stimuli such as nutrition and exercise, and accumulating evidence highlights dietary polyphenols as potent modulators of the epigenome. Through their interaction with epigenetic enzymes and signaling pathways, polyphenols may contribute to the preservation of skeletal muscle mass and function during aging, offering new avenues for the prevention and management of sarcopenia.

One of the most widely studied mechanisms is the modulation of DNA methylation. Polyphenols such as epigallocatechin gallate (EGCG), quercetin, and curcumin can influence the activity of DNA methyltransferases (DNMTs), thereby reversing aberrant methylation patterns associated with aging and muscle atrophy. By restoring appropriate gene expression profiles, these compounds may enhance mitochondrial biogenesis, antioxidant defense, and anabolic pathways essential for muscle maintenance (Ullah et al., 2020).

Importantly, evidence also shows that several polyphenols including EGCG, quercetin, curcumin and resveratrol modulate chromatin-remodeling enzymes such as DNMTs, HATs and HDACs, reinforcing their broad epigenetic impact beyond antioxidant and anti-inflammatory mechanisms (Ayissi et al., 2014).

In addition, polyphenols affect histone modifications, which regulate chromatin accessibility and transcriptional activity. Resveratrol, for example, is known to activate SIRT1, a class III histone deacetylase that orchestrates cellular stress responses, energy metabolism, and mitochondrial function. SIRT1 activation contributes to the suppression of NF-κB–mediated inflammation, enhancement of autophagy, and improvement of muscle endurance, linking resveratrol directly to pathways relevant for sarcopenia (Ayissi et al., 2014). Similarly, curcumin and quercetin modulate histone acetyltransferases and deacetylases, contributing to improved redox balance and reduced inflammaging (Cao et al., 2025).

Another critical layer of regulation involves microRNAs (miRNAs), which fine-tune gene expression post-transcriptionally. Polyphenols have been shown to modulate miRNAs involved in oxidative stress responses, insulin sensitivity, and myogenic differentiation. For instance, EGCG influences the expression of miR-133 and miR-206, both implicated in muscle regeneration and hypertrophy (Ullah et al., 2020; Arora et al., 2020). This epigenetic modulation of miRNAs is also supported by broader evidence showing polyphenol-induced changes in non-coding RNA networks (Rajendran et al., 2022).

Collectively, these findings underscore that polyphenols exert multi-layered epigenetic regulation, targeting DNA methylation, histone remodeling, and non-coding RNAs. This integrated control aligns with the geroscience perspective, where modulation of aging hallmarks such as mitochondrial dysfunction, loss of proteostasis, and cellular senescence can delay or attenuate sarcopenia. Thus, dietary polyphenols emerge not only as antioxidants or anti-inflammatory agents, but also as epigenetic regulators, positioning them as promising adjuncts in strategies to maintain muscle health during aging (Ayissi et al., 2014; Ullah et al., 2020; Cao et al., 2025; Arora et al., 2020).

## 6 Experimental evidence: polyphenols and sarcopenia

Clinical studies show that the effects of dietary polyphenols on sarcopenia are promising but heterogenous, depending on multiple factors such as polyphenols, type, dosage, intervention duration, participant characteristic, and diagnostic criteria.

The most frequently studied polyphenols were catechins (including epicatechin), isoflavones, and marine-derived oligomeric polyphenols. Catechins and Epicatechins (Tokuda and Mori, 2023; Mafi et al., 2019; Kim et al., 2013; Munguia et al., 2019) were generally associated with improved muscle strength and physical performance. For instance, Kim et al. reported enhancements in grip and knee extension strength, as well as walking speeds, after administering 540 mg of catechins for 3 months. Mafi et al., using 1 mg/kg/day of epicatechin, observed significant gains in leg and chest press strength, alongside improved performance in the timed up and go test. Regarding isoflavones, Aubertin-Leheudre et al. (Aubertin-Leheudre et al., 2007) demonstrated improvement in the muscle mass index after 24 weeks with a dosage of 70 mg/day. Chang et al. (Chang et al., 2023) reported positive effects on both muscle mass and function over 10 weeks with 87.2 mg/day, suggesting a dose-duration dependency. Marine-Derived Polyphenols were less commonly studied but indicated potential improvements in muscle strength and balance-related performance outcomes, although the sample size was limited (n = 20) (Kwon et al., 2021).

Sarcopenia was assessed via different diagnostic criteria and outcome measures, leading to heterogeneity in findings. Diagnostic criteria included EWGSOP2 (2019), Asian Working Group for Sarcopenia (AWGS), Foundation for the National Institutes of Health (FNIH), and other author-defined criteria. This variation affects comparability, as cutoff values and component emphasis (e.g., grip strength vs. gait speed) differ. Muscle mass was usually measured using appendicular skeletal muscle mass index, appendicular muscle mass index, or total muscle mass (Kim et al., 2013; Yasunobu and Mori, 2021). Most studies reported improvements or maintenance in muscle mass, particularly with longer intervention durations (>12 weeks). Muscle strength was commonly evaluated through grip strength (Tokuda and Mori, 2023; Kim et al., 2013; Kwon et al., 2021) and leg/chest press (Mafi et al., 2019). Physical performance was measured by various tests (e.g., 5-m walk, timed up and go, gait speed) (Mafi et al., 2019; Yasunobu and Mori, 2021).

Three studies (Tokuda and Mori, 2023; Mafi et al., 2019; Kim et al., 2017) included resistance or multicomponent exercise regimens, revealing that the combination of polyphenols with physical activity led to greater improvements in muscle function and performance. This supports the notion of a synergistic effect between dietary polyphenols and exercise, possibly due to enhanced muscle protein synthesis and anti-inflammatory pathways.

**FIGURE 2:**
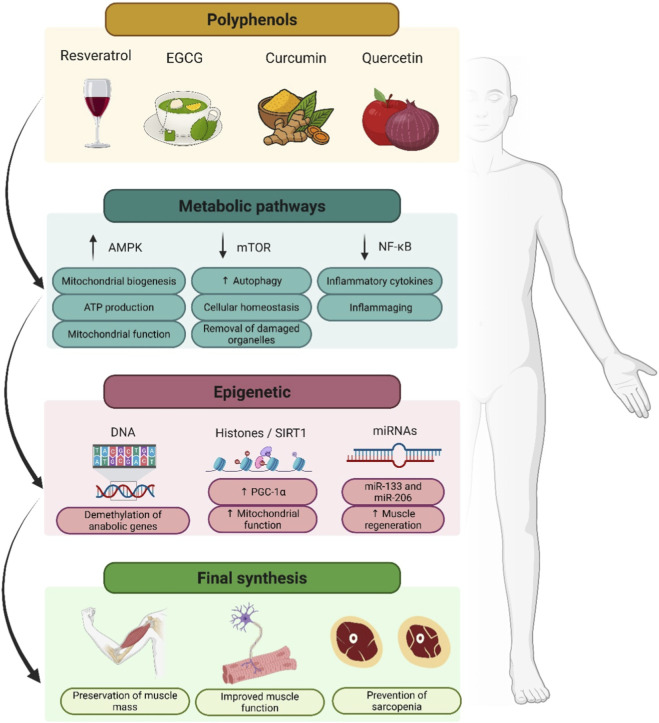
Polyphenols, molecular pathways, and epigenetic mechanisms involved in muscle health. Note - Polyphenols such as resveratrol, EGCG, curcumin, and quercetin modulate metabolic pathways (AMPK, mTOR, NF-κB), promoting mitochondrial biogenesis, autophagy, and reduced inflammation. These actions converge with epigenetic mechanisms including DNA demethylation, histone/SIRT1 regulation, and microRNAs (e.g., miR-133, miR-206) to support mitochondrial function, muscle regeneration, and the preservation of muscle mass and strength. Collectively, these effects contribute to delaying or preventing sarcopenia.

Although these clinical trials rarely investigated epigenetic endpoints, the observed functional benefits may reflect underlying mechanisms such as SIRT1 activation, DNMT inhibition, or microRNA regulation, which have been described in preclinical models of muscle aging. From a geroscience perspective, this suggests that polyphenols not only act as antioxidants or anti-inflammatory agents but also modulate key hallmarks of aging including mitochondrial dysfunction, chronic inflammation, and cellular senescence that drive sarcopenia progression. Future clinical studies should therefore incorporate biomarkers of aging, such as DNA methylation clocks and other epigenetic signatures, to better connect functional outcomes with molecular mechanisms (Figure 2).

### 6.1 Epicatechin as a modulator of myostatin signaling

Myostatin, a member of the TGF-β superfamily, is one of the most potent negative regulators of skeletal muscle growth. Its overexpression is closely associated with reduced protein synthesis, decreased satellite cell activation and enhanced proteolytic activity, contributing directly to age-related muscle atrophy. Elevated myostatin levels have been consistently reported in older adults with low muscle mass and impaired functional performance, reinforcing its relevance as a molecular hallmark of sarcopenia (Consitt and Clark, 2018).

Among dietary polyphenols, epicatechin has emerged as a promising candidate capable of modulating this pathway. Experimental work in aging mice demonstrated that epicatechin treatment reduces myostatin expression while increasing follistatin, a key physiological antagonist that promotes muscle growth. These changes were accompanied by increases in myogenic regulatory factors such as Myf5, MyoD and myogenin, suggesting enhanced regenerative potential in aged muscle (Gutierrez-Salmean et al. 2014).

In translational research, short-term administration of epicatechin in humans has produced similar patterns. A randomized trial in sarcopenic older adults found that resistance training combined with epicatechin supplementation improved muscle strength and was associated with favorable adjustments in circulating myostatin and follistatin levels (Mafi et al., 2019). Complementary evidence from clinical studies in patients with Becker muscular dystrophy also demonstrates mitochondrial adaptations and increases in follistatin following epicatechin intake, supporting the hypothesis that this compound influences molecular circuits relevant for muscle maintenance and repair (McDonald et al., 2021).

Beyond its effects on myostatin signaling, epicatechin also contributes to improved mitochondrial biogenesis and redox homeostasis, processes that decline with aging and amplify muscle atrophy. These effects are mediated through pathways such as PGC-1α activation and improved mitochondrial function, which collectively enhance muscle bioenergetics and regenerative potential (McDonald et al., 2021; Moreno-Ulloa et al., 2018).

This molecular profile positions epicatechin as a multi-target modulator capable of influencing both catabolic and anabolic signals in skeletal muscle. Its impact on myostatin–follistatin balance integrates seamlessly with the broader mechanisms discussed in this section, reinforcing the concept that polyphenols can affect muscle health not only through antioxidant or anti-inflammatory actions but also by directly modulating signaling networks central to sarcopenia.

## 7 Challenges and limitations in studies on polyphenols and sarcopenia

Although the emerging evidence points toward beneficial effects of dietary polyphenols on sarcopenia-related outcomes, some challenges and limitations hinder the ability to draw firm conclusions. These issues are largely centered around methodological variability, differences in polyphenol type and dosage, inconsistencies in outcome measurement, and a lack of rigorous long-term clinical trials.

One of the foremost challenges is the heterogeneity of polyphenolic compounds used across studies. The term “polyphenols” encompasses a vast group of bioactive compounds, including flavonoids (e.g., catechins, epicatechins, isoflavones), phenolic acids, and oligomeric polyphenols of marine origin. Some interventions used isolated compounds (e.g., pure epicatechin or isoflavones), while others employed complex mixtures, such as cocoa beverages rich in multiple flavonoids (Munguia et al., 2019). The mechanisms of action and bioavailability profiles differ substantially between compounds. For example, catechins are known for their antioxidant and mitochondrial-enhancing properties, while isoflavones have estrogenic effects, which may have sex-specific implications for muscle metabolism. The standardization and characterization of the supplements are often lacking, making it difficult to replicate findings or compare across studies. The dosage of polyphenols administered varied widely, from as little as 25 mg of epicatechin (Munguia et al., 2019) to 540 mg of catechins (Tokuda and Mori, 2023; Kim et al., 2013). This variation complicates dose-response assessments and raises questions about the minimal effective dose for clinical benefit.

Some studies used body weight-adjusted dosing (e.g., Mafi et al., 1 mg/kg/day of epicatechin), while others applied fixed daily amounts, regardless of inter-individual differences in metabolism or absorption. The intervention durations ranged from 4 to 24 weeks, often too short to detect long-term changes in muscle mass or prevent functional decline. Given the chronic and progressive nature of sarcopenia, short-term studies may only reflect transient or early-stage changes rather than durable outcomes.

Many studies were limited by small sample sizes, such as those conducted by Aubertin-Leheudre et al. (n = 18) (Aubertin-Leheudre et al., 2007) and Kwon et al. (n = 20) (Kwon et al., 2021), which reduce statistical power and increase the risk of Type II errors. In addition, there was marked heterogeneity in the diagnostic criteria used to define sarcopenia, including the EWGSOP, the AWGS, and the FNIH criteria. Not all studies assessed all three sarcopenia-related domains, muscle mass, muscle strength, and physical performance, which may lead to incomplete evaluations of intervention efficacy. Furthermore, some trials lacked methodological rigor, such as the absence of placebo controls or randomization, which increases susceptibility to bias. For example, the study by Tokuda et al. employed a single-arm design, limiting the internal validity of the findings.

Another major limitation arises from the inclusion of co-interventions that confound the interpretation of results. Several trials combined polyphenol supplementation with exercise training or amino acid co-supplementation, such as those by Tokuda (Tokuda and Mori, 2023) et al. and Mafi et al. (Mafi et al., 2019). While these multi-component approaches may better reflect real-world clinical practice, they complicate efforts to attribute observed benefits specifically to polyphenols. Additionally, many studies did not adequately control for potential confounding variables such as dietary intake, concurrent medication use, or the presence of comorbid conditions, all of which can influence muscle metabolism and interact with polyphenol absorption and bioactivity.

Given these limitations, there is a clear need for more rigorously designed, well-controlled, and long-term randomized placebo-controlled clinical trials to validate the real-world effects of polyphenols in the prevention and treatment of sarcopenia.

## 8 Geroscience perspectives and therapeutic implications

Taken together, the integration of tailored strategies based on polyphenols with structured physical exercise emerges as a promising dual-intervention approach to prevent or attenuate sarcopenia and its associated health burdens in aging populations. By targeting shared molecular pathways involved in inflammation, oxidative stress, mitochondrial dysfunction, and cellular senescence, polyphenols and exercise may have complementary effects that enhance muscle maintenance and functional capacity. By targeting inflammation, oxidative stress, mitochondrial dysfunction, and senescence, polyphenols and exercise may complement each other to preserve muscle and function.

Notably, the personalization of these strategies, considering an individual’s metabolic profile, dietary habits, and physical capacity, may enhance adherence and intervention therapeutic effectiveness. In this sense, future randomized controlled studies are warranted to confirm benefits with a focus on prevention and restoring muscle health. However, some steps are required first, as it is still necessary to determine an optimal dosage and delivery of polyphenols and establish clinically relevant biomarkers of response. Such advances could help solidify the support and role of lifestyle interventions as essential tools in combating age-related muscle decline and promoting healthy longevity.

Despite encouraging previously discussed evidence, the transition toward individualized prescriptions of polyphenol-based nutritional support and exercise interventions remains an important gap in sarcopenia research. Interindividual variability in gut microbiota composition, genetic polymorphisms, and especially epigenetic markers such as DNA methylation clocks, histone signatures, and microRNA profiles, significantly influences the bioavailability and metabolic response to polyphenols, as well as the efficacy of exercise regimens. Understanding these variables could guide the development of precision interventions to maximize therapeutic outcomes. For instance, stratifying individuals based on their ability to metabolize specific polyphenols or their responsiveness to physical training could improve intervention targeting. Further research should consider integrating omics technologies, such as nutrigenomics, metabolomics, and transcriptomics, into clinical practice will be crucial to identify biomarkers that predict treatment efficacy and guide the customization of interventions. Future trials should therefore prioritize stratified study designs and explore adaptive interventions that respond to an individual’s changing physiological profile across the aging trajectory.

## 9 Conclusion

This review highlights growing evidence that polyphenols modulate muscle aging through antioxidants, anti-inflammatory, mitochondrial, and epigenetic mechanisms. These compounds show promise as adjunct strategies for the prevention and management of sarcopenia, particularly when combined with structured physical exercise.

Despite encouraging findings, important gaps remain regarding dosing, bioavailability, interindividual variability, and long-term efficacy. Future trials should integrate biomarker validation, including epigenetic signatures, to advance precision nutrition approaches. Polyphenols within the geroscience framework underscores their potential as both modulators of aging hallmarks and regulators of muscle health, opening new avenues for translational strategies against sarcopenia.
